# Health system strengthening using a *Maximizing Engagement for Readiness and Impact (MERI) Approach*: A community case study

**DOI:** 10.3389/fpubh.2022.952213

**Published:** 2022-11-23

**Authors:** Teddy Kyomuhangi, Kimberly Manalili, Jerome Kabakyenga, Eleanor Turyakira, Dismas Matovelo, Sobia Khan, Clare Kyokushaba, Heather MacIntosh, Jennifer L. Brenner

**Affiliations:** ^1^Maternal Newborn and Child Health Institute, Mbarara University of Science and Technology, Mbarara, Uganda; ^2^Department of Community Health Sciences, Cumming School of Medicine, University of Calgary, Calgary, AB, Canada; ^3^Department of Community Health, Mbarara University of Science and Technology, Mbarara, Uganda; ^4^Department of Obstetrics and Gynecology, Catholic University of Health and Allied Sciences, Mwanza, Tanzania; ^5^The Center for Implementation, Toronto, ON, Canada; ^6^Indigenous, Local and Global Health Office, Cumming School of Medicine, University of Calgary, Calgary, AB, Canada; ^7^Department of Pediatrics, Cumming School of Medicine, University of Calgary, Calgary, AB, Canada

**Keywords:** implementation, health system strengthening (HSS), readiness, global health, engagement, maternal health, newborn health

## Abstract

**Introduction:**

Health system strengthening initiatives in low and middle-income countries are commonly hampered by limited implementation readiness. The *Maximizing Engagement for Readiness and Impact (MERI) Approach* uses a system “readiness” theory of change to address implementation obstacles. *MERI* is documented based on field experiences, incorporating best practices, and lessons learned from two decades of maternal, newborn, and child health (MNCH) programming in East Africa.

**Context:**

The *MERI Approach* is informed by four sequential and progressively larger MNCH interventions in Uganda and Tanzania. Intervention evaluations incorporating qualitative and quantitative data sources assessed health and process outcomes. Implementer, technical leader, stakeholder, and policymaker reflections on sequential experiences have enabled *MERI Approach* adaptation and documentation, using an implementation lens and an implementation science readiness theory of change.

**Key programmatic elements:**

The *MERI Approach* comprises three core components. ***MERI*** **Change Strategies** (meetings, equipping, training, mentoring) describe key activity types that build general and intervention-specific capacity to maximize and sustain intervention effectiveness. The **SOPETAR Process** **Model** (**S**can, **O**rient, **P**lan, **E**quip, **T**rain, **A**ct, **R**eflect) is a series of purposeful steps that, in sequence, drive each implementation level (district, health facility, community). A ***MERI*** **Motivational Framework** identifies foundational factors (self-reliance, collective-action, embeddedness, comprehensiveness, transparency) that motivate participants and enhance intervention adoption. Components aim to enhance implementer and system readiness while engaging broad stakeholders in capacity building activities toward health outcome goals. Activities align with government policy and programming and are embedded within existing district, health facility, and community structures.

**Discussion:**

This case study demonstrates feasibility of the *MERI Approach* to support district wide MNCH programming in two low-income countries, supportive of health outcome and health system improvements. The *MERI Approach* has potential to engage districts, health facilities, and communities toward sustainable health outcomes, addressing intervention implementation gaps for current and emerging health needs within and beyond East Africa.

## Introduction

Global commitments to the Millennium Development Goals and more recently, to the Sustainable Development Goals have prompted major investments in maternal, newborn, and child health (MNCH) interventions in low-and-middle-income countries (LMIC) (1). However, implementation challenges remain barriers to optimal intervention delivery, scale-up, and sustainability, reducing potential impact of these major investments and limiting population health outcome improvements (2).

The World Health Organization's *System Strengthening Framework* (3) is helpful in identifying needed foundational factors for functional LMIC public health systems. Common barriers within systems include lacking resources (e.g., equipment, medications, health human resources, infrastructure), weak transportation and drug distribution systems, and challenges developing and maintaining a cadre of knowledgeable and skilled workers (4, 5). Overcoming such barriers requires building capacity and health systems strengthening, long recognized by policymakers, researchers, and health programmers as core health intervention strategies. Yet ensuring that governments and development partners are actually ready to implement and receive benefit from interventions is a frequent oversight with implications for implementation quality and sustainability (6). Additional implementation gaps affecting uptake and sustainability include insufficient in-country implementer involvement (7, 8), and individual and organizational motivation for intervention adoption and ownership (6).

System strengthening frameworks and approaches primarily focus on structural challenges that may miss important implementation barriers, especially in LMICs. A comprehensive approach for stimulating and sustaining change involves consideration of “system readiness”, which posits “psychological” and “structural” preparedness for individual and organizational change (9, 10). The “psychological” aspect refers to motivation, which is key to facilitating change, but it is often overlooked. Additionally, a system that is adopting sustainable change must be ready for a *specific or general* intervention (11, 12). Shifting to a “general readiness” mindset, rather than focusing on a specific program, can strengthen overall capacity of a system to adapt to emerging needs. This is a critical and under-addressed concept in implementation work.

In this paper, we describe the history, development, and details of key features of the *Maximizing Engagement for Readiness and Impact (MERI) Approach*, a model addressing implementation gaps through incorporation of our documented best practices and lessons learned during two decades of developing, implementing, and evaluating MNCH programming in Uganda and north-western Tanzania.

## Context

### Setting

The *Maximizing Engagement for Readiness and Impact (MERI) Approach* has evolved from a series of experiences in Uganda and subsequently, Tanzania.

In Uganda, *Healthy Child Uganda* (www.healthychilduganda.org) has led implementation and evaluation since 2003. The partnership is comprised of a Ugandan university (Mbarara University of Science and Technology) and Canadian institutions, including University of Calgary and the Canadian Pediatric Society. For 20 years, *Healthy Child Uganda* has undertaken a variety of projects and programs with a common goal of improving health for pregnant women and children. These initiatives were conceptualized, planned, implemented, and evaluated by Ugandans with technical and funding support through Canadian partners. Initial projects began with small community-oriented interventions which progressively grew in scope and scale, building on lessons learned and leveraging increased funding opportunities. Populations served by MNCH programming were predominantly rural; target communities comprised of low-income families dependent on subsistence farming, experiencing difficulty accessing health facilities owing to topography, distance, and limited infrastructure. Intervention area selection favored communities with poor MNCH indicators compared to national targets.

Beginning in 2016, *Healthy Child Uganda* partners joined a coalition in Tanzania consisting of the Catholic University of Health and Allied Sciences (CUHAS), Agriteam Canada, and Save the Mothers to implement an MNCH intervention under the *Mama na Mtoto* banner. Technical and planning support was provided to the Tanzanian field team by CUHAS, Ugandans, and Canadians. A Tanzanian Principal Investigator (DM) led an effectiveness and process evaluation. Programming occurred in target communities in rural communities, predominantly farming, under-resourced, and remote characteristics albeit a different country, culture, and language context.

Uganda and Tanzania's health systems are decentralized with district health leadership managing key services and decision-making. A majority of health facilities within all intervention districts were government-owned, offering services according to facility level (13). Most districts had 1–2 hospitals with advanced care facilities providing broad inpatient and outpatient surgical and medical services; other facilities offered common and basic inpatient services, obstetrical care, and outpatient primary health care. In both countries, emerging national policy and guidelines supported a volunteer Community Health Worker (CHW) cadre intended to promote health within villages (14, 15); however, broad mobilization of this cadre had not occurred in target districts prior to intervention start.

### Initiatives and evaluation informing the *MERI Approach*

Over the past two decades, four key sequential MNCH initiatives informed the *MERI Approach*.

*MERI*-informing initiatives vary in content and context, with a general increase in scope and scale over time. Table 1 summarizes their key intervention and evaluation features.

**Table 1:** Features of key *MERI-*informing interventions, 2002-2022.

**Intervention**	***Community Owned Resource Persons (CORPs) Phase I***	***CORPs* *Phase II***	***MamaToto***	***Mama na Mtoto***	***Healthy Adolescents & Young People (HAY!)***
**Date**	2003-5	2006-9	2012-15	2016-20	2020-25
**Location**	SW Uganda	SW Uganda	SW Uganda	NW Tanzania	SW Uganda
**Coverage**	6 parishes	18 parishes	2 districts	2 districts	2 districts
**Population**	~30,000	~60,000	~350,000	~800,000	~400,000
**Focus**	Child health & nutrition	Child health & nutrition	Maternal newborn & child health	Maternal newborn health	Adolescent sexual reproductive health & rights
**Key outcomes**	Home practices	Home practices; care-seeking;	Home practices; care-seeking; U5 morbidity	Antenatal care; skilled birth attendant; postnatal care	Youth care-seeking; youth family planning
**Intervention target level**	Community	Community, facility	Community, facility, district	Community, facility, district	Community, facility, district
**Evaluation type(s)**	Operational data review; qualitative study	Prospective intervention trial (mixed methods)	Prospective intervention trial (mixed methods)	Hybrid effectiveness implementation trial	Process & effectiveness (mixed methods)
**Main outcome data source(s)**	FGD/KII	Pre-post household surveys; U5 birth/ death reports; FGD	Post household surveys; FGD/KII; facility audits;	Pre-post household surveys; FGD/KII; facility audits;	FGD/KII; facility audits; HMIS;
**Main process data source(s)**	Steering Committee (SC); core team post-project reflection	SC; formative lessons learned/best practice recording (workshops, meetings, reports); CHW motivation study; model report preparation	SC; interval reflection workshops; ext. eval.; post-project process review (incl. decision-makers); CHW registers (retention study)	RE-AIM evaluation (output indicators, KII/FGD); CIFR evaluation; ext. eval.; implementation science consultation and workshop; CHW registers	Pre/post readiness assessments; RE-AIM evaluation (output indicators, KII/FGD)

CHW, Community Health Worker; HMIS, Health Management Information System; U5, Under five (years old); ext, external; FGD, Focus Group Discussion; KII, Key Informant Interview; SC, Steering Committee; SW, southwest; NW, northwest.

Initiatives have undergone increasingly rigorous and larger scale evaluations. Mixed-method tools have been employed to assess “*effectiveness*” results. “*Process”* has been evaluated through increasingly formalized activities; reflection workshops, meetings (implementers, technical team members, stakeholders, decision-makers) and report writing informed initial model descriptions and documentation of lessons and best practices (16). Process insights subsequently influenced design of larger-scale implementation efforts. Overall, each intervention was informed by a baseline survey, dedicated project planning meetings, interval (2–4 annually) steering committee meetings (comprised of >80% country residents), stakeholder and national policymaker updates (minimum once per year), end of project evaluations, pre-post training assessments, and reflection workshops at each participant level. Focused model development and documentation workshops were held at key intervals, described below.

Our team recognized early a paucity in best practice documentation MNCH programming implementation relevant to our setting. Critical reflection, evaluation, and objective documentation of “what worked” and “what did not work” became a cornerstone of proposal planning, implementation, evaluation, and dissemination with all projects. Even where resources for formal evaluation were limited, meetings, workshops, dedicated process reporting, and broad sharing of results were prioritized. Patterns of best practices emerged and evolved irrespective of intervention location, size, or scope. Recent expertise in implementation science has enabled our *Healthy Child Uganda* team to understand, articulate, and document our implementation experiences in the context of evidence-informed frameworks.

We have shared our model, lessons learned, and best practices purposefully and widely target audiences. In-country and regional district colleagues, development partners, academic colleagues, and policymakers have been reached through demonstration site visits, study tours, end of project dissemination conferences, training workshops (short courses), national working group participation, manuscripts, abstract presentations, and open-access sharing of web-based materials including videos, summary reports, training materials, and implementation guides (www.healthychilduganda.org/materials and www.mnmtanzania.com/products-and-resources/).

#### Community Owned Resource Persons phase I (2003-5)

Our first *Healthy Child Uganda* initiated community health intervention involved 115 child health and nutrition focused CHWs in 6 communities, scattered throughout 3 southwestern Ugandan districts. Trainers from health facilities received training using programming for CHWs and government of Uganda-endorsed Community Owned Resources Persons (CORPs), which they then used to train community-selected volunteer CHWs. Health facility and *Healthy Child Uganda* project field staff jointly supervised CHW groups, who provided health promotion on home practices, encouraged care-seeking, and triaged and referred sick children to facilities.

A modest qualitative project and evaluation suggested improvements in home practices and strong CHW motivation; however, significant service gaps were identified when families with ill children sought facility-based care. Upon review with a very active steering committee comprised of district health leaders from all three participating districts, recommendations to support increased facility MNCH capacity, and increased CHW-facility linkages were made.

#### CORPs phase II (2006-9)

New funding enabled CHW network extension three-fold. To address MNCH service gaps identified from Phase I, clinical refresher workshops, CHW supervisor training and mentorship, and essential equipment (e.g., thermometers, weighing scales) provision to referral facilities was added. Increased exposure to community development concepts and opportunities for expanding income-generating projects amongst CHW groups was also enhanced, in response to growing community identification of economic instability as a root cause of health problems. During this phase, district health managers and local political leaders became increasingly engaged in the initiative.

A pre-post *CORPs-Phase II* evaluation reported significant health outcome improvements associated with CHW mobilization (17). Also during this period, volunteer CHW retention and motivation was found to be very high based on qualitative CHW data and a CHW retention assessment (18). A series of process focused key informant interviews, focus group discussions, meetings, and an end of project reflection workshop informed a detailed CORPs Model Report (16), documenting implementation steps, results, success factors, and programming recommendations.

In 2011, a sub set of CHWs from this cohort participated in a *Healthy Child Uganda*-led research study assessing CHW medication distribution through iCCM. In addition to demonstrating feasibility of iCCM by lay volunteer CHWs (19), this experience increased understanding of CHW group dynamics (20) and potential for CHW mobile phone use (21).

#### *MamaToto* (2012-2015)

A significant scale-up opportunity arose in 2012. The *MamaToto* (mother-child in Swahili) initiative enabled MNCH programming throughout two full Ugandan districts including strengthening of a 2,700-member strong CHW network. This enabled full district coverage, with potential to test increased district implementation leadership and overcome challenges faced by piece-meal intervention coverage. Leadership within intervention districts was motivated and heavily engaged in planning; with an eye toward sustainability and strengthening of existing structures, additional capacity was built amongst health managers, data clerks, and facility governance committee members and reflection processes were formalized at all levels of participant engagement.

A comprehensive pre-post household survey and associated qualitive evaluation at endline documented effectiveness in improving MNCH outcomes; high CHW retention was again documented amongst this larger cohort (22). Volunteer motivation (23) and the importance of supportive supervision (23) were articulated. Increased district-level engagement and a high-degree of embeddedness within existing systems were credited for promoting sustainability.

At intervention end, national policymakers as well as district leaders from 4 East African countries participated in a symposium including field excursions. Two national leaders, the lead district health manager and 5 authors (TK, KM, HM, JK, and JB) participated in a 5-days workshop, culminating in a refined best practice document and a draft process model (SOPETAR, described below). In 2018, *MamaToto* was recognized as a top Ugandan social innovation in health, enabling subsequent longitudinal mentorship from a Ugandan panel to strengthen documentation.

#### *Mama na Mtoto* (2016-20)

An opportunity to scale-up (replication) implementation of *MamaToto* experiences in a new country setting (Tanzania) arose in 2017 following participation in the 2015 *MamaToto* symposium by Tanzanian district leaders. Named *Mama na Mtoto* (Swahili for mother-baby*)* by intervention communities, this initiative enabled MNCH programming by a coalition of partners in 2 districts in Lake Zone, Tanzania. Targeting the largest population to date (~800,000), more than 1,660 CHWs and 480 health workers were trained. Implementation mirrored Ugandan *MamaToto* processes adapted for a new setting and implementation team.

A comprehensive process and effectiveness evaluation (24) was enabled through a separate research grant. Full district replication of *MamaToto* proved feasible with minimal modifications to accommodate setting, health system differences, and national guidelines/policy. Based on a pre-post household survey, key pre-identified MNCH indicators were significantly improved (25). A process evaluation included interval monitoring using the RE-AIM framework (26) and an implementation assessment using the Consolidated Framework for Implementation Research (27), which captured feedback from implementers, technical team members, and key stakeholders. Findings from Ugandan and Tanzanian experiences were shared with a Canadian implementation science expert (SK) who subsequently facilitated a refined blueprint and feedback from all authors, including through a 3 day in person workshop with project leads (TK, KM, HM, SK, and JB). Subsequently in person and in-depth meetings to refine a final document with all authors and later other stakeholder groups were conducted.

#### Healthy adolescents and young people (HAY!) (2020-2025)

The *MERI Approach* is currently used to guide the *Healthy Adolescents and Young People (HAY!)* initiative which builds on past intervention lessons and adapts them to a new content area of adolescent SRHR, across two full Ugandan districts. Like past MNCH interventions, the *HAY!* initiative involves district engagement, health facility strengthening, and CHW mobilization within this new content focus area. Since activities, policies, and practices influencing adolescent health and wellness involve a variety of sectors (i.e., health, education, justice, economic development) and macro-level considerations, MERI strategies to promote multi-sectoral engagement and readiness are being trialed and evaluated.

## Key programmatic components

### Theory of change *(R = MC^2^)*

The *MERI Approach* comprises several components that together optimize intervention implementation. It incorporates real-world best practices, carefully evaluated to document effectiveness with a focus on fostering readiness, health system strengthening, and sustainability. In articulating the *MERI Approach*, we adopted “*R* = *MC*^2^” as our theory of change (28). Scaccia et al. (28) describe intervention-specific and general readiness concepts in this simple heuristic where *readiness* (R) is a product of *motivation* (M), *intervention-specific capacity* (C), and *general capacity* (C). In this comprehensive, trans-disciplinary readiness framework, a motivated system is one in which system actors are willing to adopt an intervention and to innovate in general. *Intervention-specific capacity* refers to capabilities and structures that are in place for a particular intervention. *General capacity* refers to general characteristics of the system that fosters overall readiness for implementation. *Readiness* is the product of these components rather than the sum, because if any one of these is zero, readiness cannot be achieved.

### Core components

Guided by a system readiness theory for change, the *MERI Approach* uses three core components: (1) key change strategies, (2) a process model to guide the activity sequence, and (3) a motivational framework, comprising factors that promote stakeholder buy-in and sustainability.

#### MERI Change Strategies (R = MC^2^)

The program theory describes two levels of intervention and implementation strategies (28). At the first level, “general” capacity and motivation for change are addressed within the implementation setting (in our case, at district, health facility, and community levels). The second level relates to the intervention-specific capacity where motivation is required for intervention-related outcomes. Four key *MERI* Change Strategies address common implementation capacity barriers while leveraging existing opportunities (e.g., ongoing district activities) to promote “general capacity” and “intervention-specific capacity” (see examples in Table 2): collaborative and consensus building meetings, equipping, training, and technical assistance and mentorship. Additional emerging change strategies may be added according to implementation science based on barrier/facilitator assessments and stakeholder priorities for a given intervention.

**Table 2:** *MERI* change strategy activity examples: General and intervention-specific capacity.

**Strategy**	**Examples**	
	***General capacity***	***Intervention-specific capacity***
**Meetings**	Gathering community members to develop a community-level action plan to improve resident health.	Gathering district health managers to prepare a maternal, newborn, and child health focused strategic plan.
**Equipping**	Providing a district with an ambulance to support emergency referral.	Providing each health facility with a bag and mask device to enable newborn resuscitation.
**Training**	Conducting a workshop for facility data managers to practice health management information analysis and graph preparation.	Conducting a simulation training workshop for clinical staff to practice case management of obstructed labor.
**Mentorship**	Facilitating a visit by a district health manager to a primary health facility to review patient triage and referral practices.	Facilitating a visit by a regional hospital pediatrician to a primary health facility to review recent sick child case management.

#### The Scan, Orient, Plan, Equip, Train, Act, Reflect (SOPETAR) process model

The SOPETAR (**S**can, **O**rient, **P**lan, **E**quip, **T**rain, **A**ct, **R**eflect) sequence guides the *MERI Approach* implementation steps at district, health facility, and community levels through an iterative implementation cycle. As a *process model* (29), SOPETAR describes implementation order and operationalization (“how-to”); for *MERI*, this includes a comprehensive description of change strategy rollout. SOPETAR (Table 3) has elements similar to other implementation process models [e.g., *Getting to Outcomes* (30)] and quality improvement cycles (31); our team members have background in both disciplines and project activities draw on both fields (32). While on a surface level SOPETAR steps are common amongst community development and health system strengthening initiatives, their quality and order are often not carefully considered, leading to sub-optimal implementation. Based on reflection (participant, stakeholder, implementation team), we describe SOPETAR best practices, drawing on lessons learned and programming successes to mitigate common implementation gaps and maximize participant engagement at each level. SOPETAR steps are purposeful, timed, and ordered, requiring specific considerations to promote implementation quality. Initially, at each level, participants are supported to learn the process as they move through the SOPETAR cycle. Once learned, participants apply SOPETAR process steps themselves as they plan, implement, and sustain change strategies.

**Table 3:** Scan-Orient-Plan-Equip-Train-Act-Reflect (SOPETAR) process model steps.

**Step**	**Details**
**S**can	Increases understanding by implementers and stakeholders of available resources, personnel, structures, and relevant policy to steer strategy, operations, context and gaps; involves appropriate stakeholders, promotes embeddedness. Scan data is incorporated into subsequent steps, enhancing efficient and focused planning, equipping, training, and action.
**O**rient	Promotes early, purposeful, clear introduction about the upcoming intervention; encourages broad, sequential (i.e., cascade), and purposeful stakeholder engagement. Guided by the principle of collective action, orientations are conducted by trusted individuals, encouraging participation, and providing early opportunity to voice concerns and ideas at all levels.
**P**lan	Considers strengths, gaps, and priorities prior to equipping, training and actions; ensures participant contributions to clearly documented and agreed-upon actions and targets. Planning meetings occur with key stakeholder groups at all levels and sites, following a specific, dedicated, and focused process; alignment with existing systems, policies, and planning processes is encouraged.
**E**quip	Ensures necessary materials, supplies, and equipment for participant group to complete quality training and put new skills into action. Equipment is provided according to stakeholder priorities during planning. An equipment orientation and maintenance strategy is critical to optimizing use and longevity.
**T**rain	Builds general and specific capacity at all levels. Training workshops engage using participatory and train-the-trainer approaches. Workshops occur within communities, use a low-cost model, are consistent with national programming; regular refreshers promote retention and sustainability.
**A**ct	Actions follow a workplan guiding quality activities at all implementation levels to reach ultimate beneficiaries. Innovation and use of existing resources are encouraged. Action plans are monitored and revised regularly.
**R**eflect	Enables key user groups to identify, consider, and address implementation challenges, successes, facilitators, and barriers. This reinvigorates the SOPETAR cycle and its steps for the subsequent sustainability phase.

To foster greater readiness, SOPETAR increases emphasis on exploration and preparatory implementation phases, especially SCAN, ORIENT, and PLAN. Our experience has demonstrated improved implementation success due to substantial time and resources toward broad engagement in these stages. Participant groups engage in **SCAN** activities to gain capacity to identify local needs and resources, thus focusing the intervention. This phase may complement a usual baseline study, but it includes a targeted assessment of existing resources, understanding of common practices, and is conducted with significant participation from stakeholders and decision-makers. Next, broad and purposeful **ORIENTATION** is conducted using a “cascade” approach, with one level orienting the next, promoting buy-in and motivation. During **PLANNING**, all participants develop intervention specific content area action plans relevant to their local needs. Often, low-income settings lack equipment and supplies, posing very significant barriers to implementation (5). Unlike process models developed primarily for high income settings, SOPETAR's **EQUIP** is drawn out as a specific process step and conducted early to ensure sites can conduct subsequent steps effectively, especially ensuring equipment is available prior to training and enables use of newly acquired skills and knowledge. **TRAINING** involves increasing skills and knowledge through “train the trainer” (i.e., build cascading capacity for individuals to train others across levels of the system). Participants then enact their action plans during **ACT**, conducting innovative and relevant activities within their expected roles. Finally, during **REFLECTION**, intervention challenges and opportunities are noted and discussed using a formal, interactive discussion process with each participant group. In subsequent implementation cycles, participant groups can revisit relevant SOPETAR steps as needed.

#### *MERI* motivational framework

The third *MERI* component includes factors to maximize motivation, engagement, and sustainability. While *MERI Change Strategies* address general and intervention capacity gaps, motivational challenges (i.e., being willing and psychologically “ready”) is another crucial aspect of system readiness (28). We consistently identify five core “motivational” factors (described in Table 4) cross-cutting various settings at all levels: self-reliance, collective action, embeddedness, transparency, and comprehensiveness. These factors stimulate positive intervention perceptions (33, 34) and promote self-determination (35).

**Table 4:** Foundational factors in the *MERI* motivational framework.

**Factor**	**Description**
**Self-reliance**	Districts, health facilities, and communities are encouraged to create change by themselves for themselves. Autonomy is promoted at all levels, cultivating innovation, local solutions for local needs, and resourcefulness through a “use what you have” philosophy. Participatory facilitation methods grounded in community development theory encourage skill development, meaningful dialogue, and critical thinking while discouraging dependency.
**Collective action**	Broad, informed, and action-oriented engagement cultivates constructive relationships and cooperation. A culture of “everyoneness”, investment in champions, and a clear and unified goal enhances connectivity and momentum within and between levels. Resolve for change is affirmed and drives collaborative implementation and action.
**Embeddedness**	Maximizing compatibility with district and community priorities, structures, and processes is integral to change implementation. Existing resources and people are leveraged; activities align with district and national programs and policies. Investment in district leadership as key implementation team members reinforces use of established structures and reporting relationships. A “cascade approach” sees initial activities amongst district stakeholders, who, in turn, support health facility capacity development, whose representatives then engage communities.
**Transparency**	Clear and consistent communication and practices from implementation outset proactively address common expectation gaps. Well-documented implementer, participant, and beneficiary roles and responsibilities are shared early and widely; problems are addressed quickly and openly.
**Comprehensiveness**	A “whole system” approach ensures intervention compatibility with all structures (health and non-health) throughout an entire district. District-wide and broad coverage, reach, and comprehensive engagement seek to leave none out, consistent with district health system priorities.

### Bringing it all together

**Figure 1:**
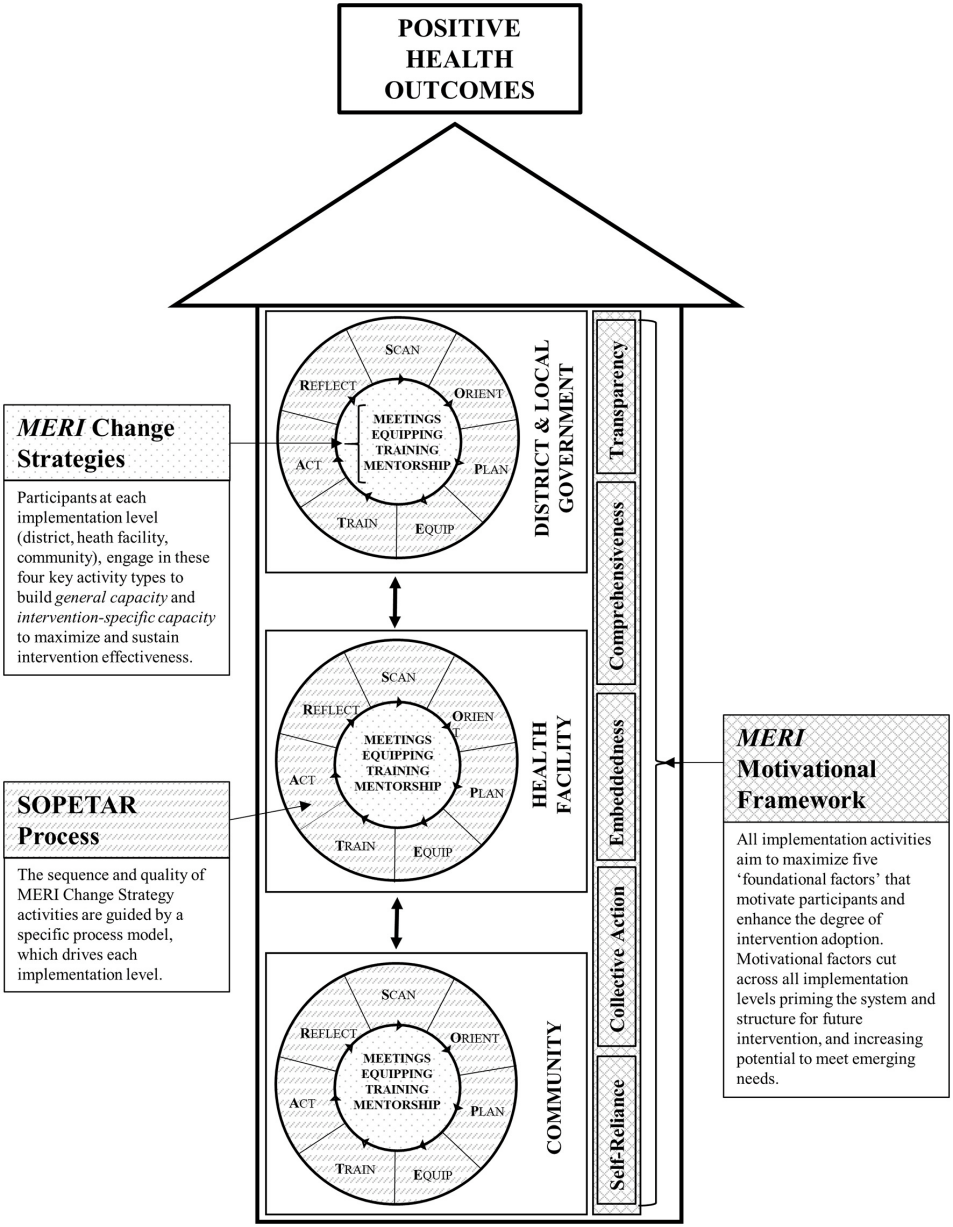
How *MERI Approach* components work together.

Figure 1 illustrates how the three *MERI Approach* components work together. *MERI Change Strategies*, the SOPETAR process model, and a *MERI Motivational Framework* are integrated at all implementation levels. At each level, the SOPETAR process guides the sequence and quality of change strategies which foster both intervention specific capacity and general capacity. Motivational factors permeate all activities at all levels.

## Discussion

The *MERI Approach* with its system readiness lens is informed by our experiences and implementation science theory. Using Scaccia et al. (28) *R* = *MC*^2^ framework (28) as a theory of change, *MERI* illustrates an approach “by implementers for implementers”; its three components work together optimizing intervention implementation. *MERI* components incorporate real-world best practices and lessons learned, rigorously evaluated and purposefully refined in two countries. Feasible for full district implementation, the *MERI Approach* is designed to cultivate *readiness* while fostering improved “specific” health outcomes, strengthening health systems overall and promoting sustainability.

As a health system strengthening-directed approach, *MERI* activities target outcome changes at multiple levels at a time (i.e., district, health facility, community). This whole-system, “population health approach” (36, 37) acknowledges that health change is accelerated when barriers are addressed at all levels and when a health system can support all system actors (leaders, managers, providers, communities, and districts) without gaps, to implement health interventions. However, beyond being a health system strengthening approach, the *MERI Approach* additionally applies a system readiness for implementation lens, building “general system” readiness while increasing “intervention-specific” readiness for change. Improved readiness creates a context more prepared for further and future implementation efforts enabling health systems to meet emerging needs.

Based on our own practical experience and observations in Uganda and Tanzania, health intervention efforts frequently assume that systems, organizations, and communities are ready and willing (i.e., have capacity and motivation) to implement evidence-based interventions. Yet, we have often seen where core capacity or motivation (or lack of) by governments, districts, and communities is seemingly overlooked. In a review of organizational readiness tools for global health interventions, Dearing (6), argues that in low-income contexts, assessing motivation for intervention delivery is particularly critical; organizations (or systems or communities) are usually addressing multiple competing and often, urgent, challenges, and as such, some interventions will be consigned as lower priorities. One unique *MERI* aspect is articulating foundational factors that directly motivate and understand motivation of participants, invest in orientation to promote early buy-in, maximize engagement, and draw on existing assets, processes, and structures, which is particularly valuable in a resource constrained setting. *MERI* Motivational Framework factors are consistent with self-determination theory (relatedness, competence, autonomy) (35) and evidence-based implementation factors described in the Consolidated Framework for Implementation Research (compatibility, relative advantage and priority, leadership engagement) (33).

Through its processes, the *MERI Approach* addresses critical gaps (6, 38) in attention to sustainability factors. For example, the SOPETAR process model fosters local leadership, ownership, and priority through active and repeated stakeholder practice of core implementation skills (planning, monitoring, and reflection) during the project cycle. That *MERI* Change Strategy activities are conducted by district health leaders, trainers, and champions, promoting alignment with district long-term strategy and broadening overall district implementation capacity. Furthermore, *MERI* Motivational Framework factors mitigate commonly cited sustainability challenges through self-reliance, encouraging existing resource use, and reducing longer-term programming costs and dependence risk (7).

## Conceptual and methodological constraints

We will continue to refine the *MERI Approach* to overcome challenges in adoption and uptake. Full *MERI* Implementation requires a significant commitment to quality, detail, and specific activity order requirements. However, based on our own experiences, SOPETAR steps and application across all levels and entire districts are key for maximum impact. For example, extensive time and resource investment in orientation activities within our first *Mama na Mtoto* district in Tanzania initially met with hesitation by funders, government officials, and implementation team members accustomed to more rapid start up. However, by implementation start in the second intervention district, the purposeful and intense orientation process was better understood and its quality and added value appreciated. We learned that ensuring good understanding of the *MERI Approach* and its rationale by implementation teams and stakeholders is critical to implementation success. Additionally, time and resources intense steps during and after the intervention are balanced by utilizing available human and tangible resources within existing systems, reducing long-term maintenance costs for embedded activities.

*MERI's* very structured process may be challenging to implement in certain contexts. For example, flexibility in timing maybe limited when dealing with humanitarian situations and critical deliverables. However, based on our recent adolescent SRHR programming experiences during an unpredictable novel coronavirus-19 pandemic, general commitment to *MERI* principles including quality and order can still be feasible. If readiness, especially motivation, is truly stimulated, there are dividends in district health systems who, with a strong foundation, can pivot to meet emerging, unpredicted needs without significant external support.

**Figure 2:**
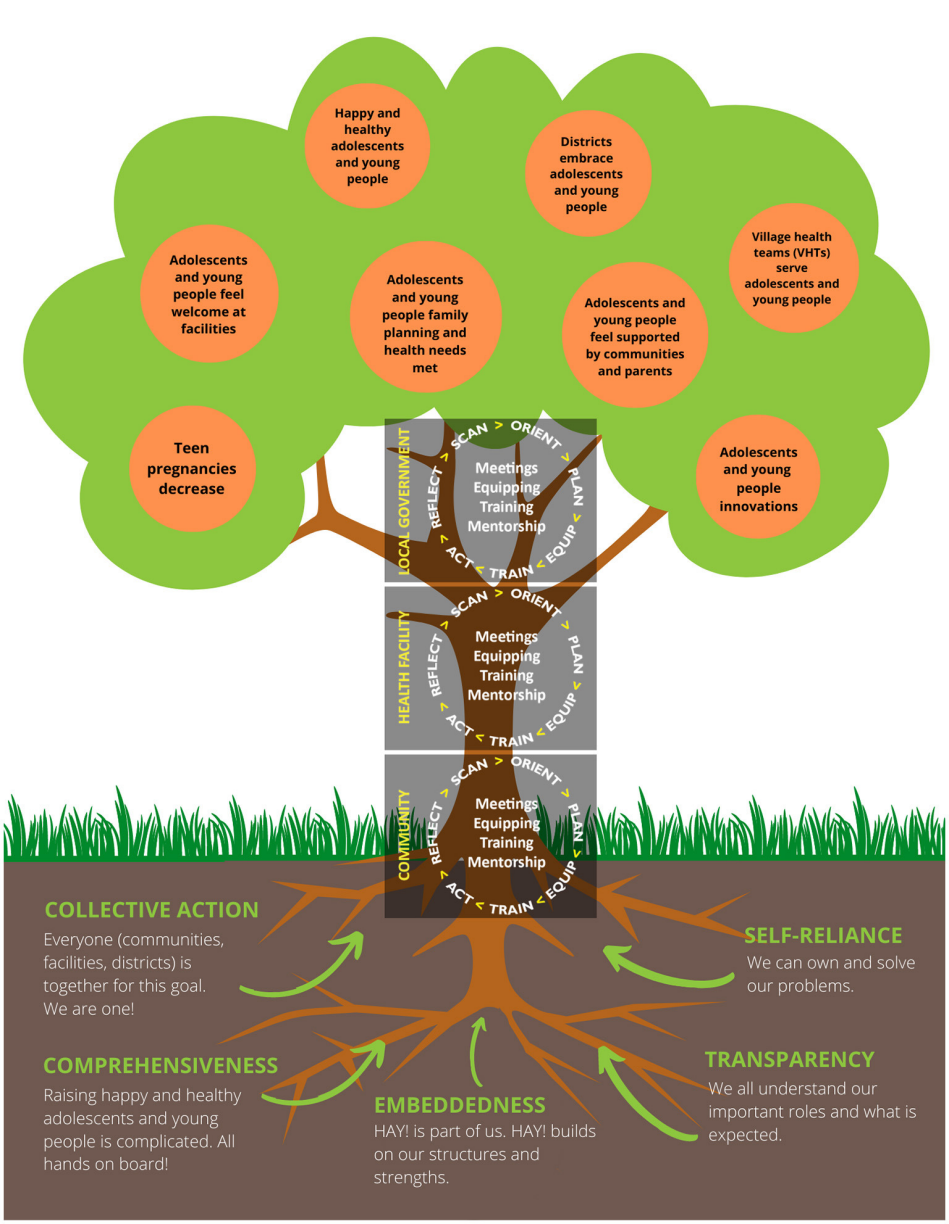
*MERI Approach* tree (community version).

Packaging the *MERI* model requires thoughtfulness, creativity, and adaptation for context. Implementers require early and in-depth orientation and guidance, whilst integrating their own valuable experiences and expertise. This requires dedicated time and investment. At the community level, explaining a complicated “implementation science” process may seem improbable, but with adaptation, it can be achieved and effective. Recently with local stakeholders, a modified community-friendly version of the *MERI Approach* was developed, using a widely understood analogy of a fruit tree (Figure 2). This image is posted and discussed at the orientation sessions with every participant group during our current *HAY!* initiative. It generates conversation and common understanding about roles, expectations, and priorities amongst stakeholders and beneficiaries, regardless of role or literacy level, prior to activity implementation. Another example of adaptation for broad implementation engagement occurs during “plan” sessions where different stakeholder groups use a “rose” and “thorn” activity (i.e., rose = facilitator; thorn = barrier) to identify locally relevant barriers and enablers.

In both Tanzania and Uganda implementation delivery teams use English during meetings and plan to have access to paper handouts and computer-assisted presentations. In contrast, in the community context, sharing of implementation concepts and tools (adapted *HAY! Tree* “rose/thorn” activity mentioned above) often requires translation into local dialect, visual representation of concepts, and posting on locally available materials (e.g., rice bags) to accommodate audience literacy, language, and venue. At all levels, participants show interest in engaging and understanding implementation concepts. Within our current Ugandan adolescent health initiative, such adaptations are overseen by a dedicated “implementation coaching team” whose members mentor implementing district leaders, monitor processes, assess implementation strength and progress, and identify and address emerging implementation gaps. Additionally, this team considers incorporation and articulation of macro-level factor management, especially multi-sectoral engagement within *MERI*, which is critical in adolescent health and wellness.

Further research and documentation opportunities include comprehensive *MERI* evaluation within a full project cycle. To date *a priori* evaluation has only occurred for the SOPETAR model and *MERI* Change Strategies; the *MERI* Motivational Framework was articulated and added following the *Mama na Mtoto* intervention and its fuller evaluation is pending. Additionally, the extent of readiness to change amongst stakeholder groups has yet to be prospectively documented. The *MERI Approach* also warrants further testing in settings where there are no prior established relationships. In our settings, in-country institutional partners (universities) had clinical working relationships with district health leaders prior to implementation start. What additional *MERI* adjustments might be required for success where no prior relationship exists or at the national scale-up level?

## Conclusion

Critical evaluation and reflection on a series of MNCH implementation initiatives in East Africa provide insights about the process and value of strengthening health system readiness. “Readiness”, incorporating motivation, general capacity, and intervention specific capacity at all levels is crucial for early and longer-term health intervention impact. Further research to assess broader *MERI Approach* application in different contexts is warranted, such as beyond East Africa, in higher-income settings, in communities with no prior established relationship, and for national level scale-up. Informed by nearly two decades of health programming experience in complex low-income systems, the *MERI Approach* encourages readiness with demonstrated health impact and potential for health systems strengthening in preparation for emerging and dynamic health challenges within and beyond East Africa. Development partners, policymakers, funders, and implementation researchers, especially those looking to make a difference in low-income settings, can learn from our experiences and adapt the *MERI Approach* in part or in full toward greater and sustainable change and lives saved globally.

## Data availability statement

The original contributions presented in the study are included in the article/supplementary material, further inquiries can be directed to the corresponding author.

## Author contributions

JK, TK, and JB initially conceptualized and initiated best practice analyses and documentation. All authors were involved in planning and implementing one or more case study initiatives. JK, TK, JB, ET, DM, HM, CK, and KM developed and led evaluation, reflection and dissemination activities that informed this model. All authors participated in analysis and review of results and contributed to model design. SK and KM strengthened integration within implementation science frameworks. SK, TK, KM, and JB drafted the initial manuscript. All authors contributed to final manuscript review and revision.

## Funding

Implementation described herein received primary financial support from the Government of Canada through Global Affairs Canada or equivalent departments during the past two decades; workshops for model documentation and manuscript preparation were funded through an Innovating for Maternal and Child Health in Africa (IMCHA; #108024-001). IMCHA is a partnership between Global Affairs Canada (GAC), the Canadian Institutes of Health Research (CIHR), and Canada's International Development Research Centre (IDRC).

## Conflict of interest

The authors declare that the research was conducted in the absence of any commercial or financial relationships that could be construed as a potential conflict of interest.

## Publisher's note

All claims expressed in this article are solely those of the authors and do not necessarily represent those of their affiliated organizations, or those of the publisher, the editors and the reviewers. Any product that may be evaluated in this article, or claim that may be made by its manufacturer, is not guaranteed or endorsed by the publisher.

## Author disclaimer

The views expressed herein do not represent those of any funders.

## Abbreviations

CHW: Community health worker
HCU: Healthy Child Uganda
LMIC: Low-and-middle-income countries
MDG: Millennium Development Goals
*MERI*: *Maximizing Engagement for Readiness and Impact*
MNCH: Maternal, newborn and child health
SRHR: Sexual reproductive health and rights.
